# Adoption of additive manufacturing in oral and maxillofacial surgery among university and non-university hospitals in Sweden: findings from a nationwide survey

**DOI:** 10.1007/s10006-023-01147-5

**Published:** 2023-03-15

**Authors:** Xuewei Zheng, Ruilin Wang, Anders Brantnell, Andreas Thor

**Affiliations:** 1 Department of Civil and Industrial Engineering, Industrial Engineering and Management, Ångströmlaboratoriet, Uppsala University, Lägerhyddsvägen 1, 752 37 Uppsala, Sweden; 2 Department of Women’s and Children’s Health, Healthcare Sciences and E-Health, Uppsala University, MTC-Huset, Dag Hammarskjölds Väg 14B, 1 Tr, 752 37 Uppsala, Sweden; 3 Department of Surgical Sciences, Plastic & Oral and Maxillofacial Surgery, Uppsala University, Akademiska Sjukhuset, Ingång 79, 751 85 Uppsala, SV Sweden

**Keywords:** Additive manufacturing, 3D printing, Oral and maxillofacial surgery, Adoption, Survey

## Abstract

**Purpose:**

Additive manufacturing (AM) is an innovative printing technology that can manufacture 3-dimensional solid objects by adding layers of material from model data. AM in oral and maxillofacial surgery (OMFS) provides several clinical applications such as surgical guides and implants. However, the adoption of AM in OMFS is not well covered. The purpose was to study the adoption of AM in OMFS in university and non-university hospitals in Sweden. Three research questions were addressed: What is the degree of using AM solutions in university and non-university hospitals?; What are AM solutions used?; How are the AM solutions accessed (production mode) in university hospitals and non-university hospitals?

**Methods:**

A survey was distributed to OMF surgeons in Sweden. The questionnaire consisted of 16 questions. Data were analyzed through descriptive and content analysis.

**Results:**

A total of 14 university and non-university hospitals were captured. All 14 hospitals have adopted AM technology and 11 of the hospitals adopted AM in OMFS. Orthognathic and trauma surgery are two major types of surgery that involve AM technology where material extrusion and vat polymerization are the two most used AM technologies in OMFS. The primary application of AM was in medical models and guides.

**Conclusion:**

Majority of Swedish university hospitals and non-university hospitals have adopted AM in OMFS. The type of hospital (university or non-university hospital) has no impact on AM adoption. AM in OMFS in Sweden can be perceived to be a mature clinical application.

**Supplementary Information:**

The online version contains supplementary material available at 10.1007/s10006-023-01147-5.

## Introduction

Additive manufacturing (AM), also known as 3D printing or additive layer manufacturing, fabricates components by creating continuous cross-sectional layers of an object. Different from traditional subtractive manufacturing, AM uses energy to melt, fusion, and agglutinate materials layer by layer according to a computer-aided design (CAD) file, offering abilities to build parts with greater geometric complexity and a more extensive range of material selections [1, 2]. AM has spread to most clinical applications and is considered a cornerstone of the cybernetic revolution [3]. Although the technology is not yet fully realized in all clinical areas, recent advances in AM enable on-demand and patient-specific treatments, especially in oral and maxillofacial surgery (OMFS). More than 50% of the 3D printed clinical applications focus on medical equipment in OMFS [4, 5]. AM is currently being extensively studied for various surgical applications, such as biomedical materials, surgical equipment, and maxillofacial implants [4, 6–8]. Furthermore, the technology is applied to many dental and surgical cases, such as restorations, alloplastic patient-specific reconstruction, and regeneration of jaw tissues.

There are five AM methods specifically with potential applications in OMFS: stereo-lithography (SLA), selective laser sintering (SLS), fused deposition modeling (FDM), photopolymer jetting (PPJ), and powder binder printing (PBP) [9]. These methods are applicable to four main purposes for using AM in OMFS, including medical models, surgical guides, implants, and bioprinting [6]. Firstly, Nicot et al. [10] state that craniofacial anatomical models for teaching and training surgeons facilitate communication among health professionals and between doctors and patients and improve accuracy during surgery [10, 11]. 3D printing models could be used for all kinds of surgical bone plates, reducing the operation time and promoting recovery [12]. Secondly, it is estimated that the most frequent use of AM in OMFS consists of surgical guides, mainly for preoperative planning, using SLA [12]. Surgeons could for instance print a model of a defect jaw before operation and through this practice and prepare for the surgery [13]. Thirdly, 3D printed implants and prostheses have a wide range of usage in OMFS. Using SLS and PBP for making maxillofacial implants is more intuitive, time-saving, and labor-saving, with better esthetic symmetry and functionality in the mandible and craniomaxillofacial region [14, 15]. A newly developed material, polyether ether ketone (PEEK), has become a widely used material for making surgical implants and prostheses and shows promising results in outcome studies [4]. Lastly, bioprinting in multilayered skin, bone, vascular grafts, tracheal splints, heart tissue, and cartilaginous structures are examples of 3D bioprinting [16]. However, Tayebi et al. [9] state that bioprinting may not be an applicable solution to OMFS in the near future due to the unusual physical characteristics of the material, transportation difficulty, lack of formulations of hydrogels, and growth of the cells [9].

Although there is considerable research on the possible benefits and clinical applications of AM in OMFS, relatively little is known about the adoption of AM in this area. A nationwide survey investigating the adoption of AM in OMFS in Germany (*n* = 156) among university and non-university hospitals showed a relatively high AM adoption rate of 62.2%, with university hospitals more likely to adopt AM [17]. Both adopters and non-adopters were generally positive towards AM [17]. Another study focusing on AM adoption in the clinical use of head and neck area in Finland among university hospitals (*n* = 58) found that majority of the hospitals have adopted AM (59%): 23% said they had used AM, and 36% showed a positive attitude towards using AM [18]. Previous studies have clearly shown that OMFS and dental surgery are spearheading AM adoption among clinical use areas, but at the same time, few studies focus on actual clinical adoption. The lack of studies on adoption entails a lack of evidence-based knowledge to facilitate the adoption of AM in OMFS. To facilitate adoption is vital to realize the potential of AM in OMFS. This study aims to address the adoption of AM in OMFS in Sweden. Sweden is an innovative country that invests 3.4% of its GDP in R&D, and in 2021 it was ranked as the second most innovative country in the world according to World Intellectual Property Organization [19, 20]. To this end, the adoption of AM in OMFS in Sweden is expected to be at parity with adoption in other OECD countries such as Germany. Successful adoption examples can provide guidance for other OECD countries interested in adopting AM in OMFS. This study will address three research questions: What is the degree of using AM solutions in university and non-university hospitals? What are AM solutions used? How are the AM solutions accessed (production mode) in university hospitals and non-university hospitals?

## Materials and methods

A questionnaire was developed and administered through the online software SurveyMonkey, and data analysis was performed by using IBM SPSS statistic, version 20 (Armonk, NY, IBM Corp).

### Research design

Quantitative survey design was employed to conduct the study. The questionnaire consisted of a set of background questions (Q1–Q8), such as surgical specialty and years in office, multiple-choice questions (Q9–Q15), and an open-ended question (Q16) (for details see Supplementary file 1).

### Target population and procedure

The survey was distributed through an e-mail contact list (*n* = 348) provided by the Association of Oral and Maxillofacial in Sweden. The contact list also included personnel other than OMF specialists, including equipment suppliers and AM specialists in the field of OMF. The purpose was to capture OMF surgeons under training (ST OMF surgeons) and specialized OMF surgeons. According to the Association of Oral and Maxillofacial Surgeons in Sweden (AOMFS), the entire population of OMF surgeons (Käkkirurgi) in Sweden is *n* = 171. Of this population, our expert estimation (one of the authors is a leading OMF surgeon in Sweden) was that a subset of this is OMF surgeons under training and specialized OMF surgeons (*n* = 90). The survey was sent out by e-mail to the respondents in April 2022, with two follow-ups during April and May 2022.

### Data analysis

Descriptive analysis and content analysis were used to analyze the collected data. Inferential analysis was conducted to address the adoption rate of AM. An independent sample *T*-test was used to investigate whether the university type (university vs. non-university hospital) has a significant impact on the adoption of AM (*p* value of 0.05 was used).

## Results

The survey was e-mailed to the respondents in April and May 2022. The e-mail bounce rate was 12.4%, making 305 successful deliveries. After removing answers that did not meet the inclusion criteria (i.e., specialist under training or specialist OMF surgeon), a total of 31 participants remained. Given the evaluated population size of 90, the response rate was 34%. Out of 31 responses, all respondents answered Q1–Q8, 29 answered Q9–Q13, 18 answered Q14–Q15, and 13 answered Q16 as shown in Supplementary file 1. Since it was not mandatory to answer all questions, partial data on some items was obtained.

Of all 31 respondents, 20 (64.51%) are positioned in university hospitals, and 11 (35.49%) work for non-university hospitals. The respondents were between 35 and 54 years old (77.42%) and male surgeons (83.87%). Representative for respondents is Doctor of Dental Surgery (DDS) title (61.29%) and position as an OMF specialist (67.74%). For details on respondent characteristics see Table 1. Out of 29 surgeons who responded, 19 (65.5%) had experience with AM technology, and 10 (34.5%) had never used AM technology before. Out of 19 surgeons who had experience, 12 of the respondents were from university hospitals (Table 2).

**Table 1:** Respondent characteristics (*n* = 31)

Question	Response categories	Responses	
		%	*N*
Age	35–44	35.48%	11
	45–54	41.94%	13
	55–64	19.35%	6
	75 or older	3.23%	1
Gender	Male	83.87%	26
	Female	16.13%	5
Workplace	University hospital	64.51%	20
	Non-university hospital	35.49%	11
Highest academic title	DDS^a^	61.29%	19
	DDS and MD	12.90%	4
	PhD	12.90%	4
	Associate Professor	9.68%	3
	Professor	3.23%	1
Current position in the hospital	Specialist Oral and Maxillofacial Surgery and DDS	67.74%	21
	Specialist Oral and Maxillofacial Surgery, DDS, and MD	12.90%	4
	Resident (ST) Oral and Maxillofacial Surgery and DDS	12.90%	4
	Resident (ST) Oral and Maxillofacial Surgery, DDS, and MD	3.23%	1
	Others	3.23%	1
Surgery done most by the respondent monthly^b^	Dento-alveolar surgery	48.39%	15
	Dental implant surgery	48.39%	15
	Bone grafts and replacement	25.81%	8
	Orthognathic surgery	45.16%	14
	Maxillofacial trauma	48.39%	15
	TMJ surgery	19.35%	6
	Pathology and reconstruction	32.26%	10
	Facial cosmetic surgery	6.45%	2
	Others	9.68%	3

^a^DDS stands for Doctor of Dental Surgery

^b^The percentages go beyond 100% due to multiple answers per respondent

**Table 2:** Surgeons’ experience of AM (*n* = 29)

	Respondents from university hospitals	Respondents from non-university hospitals	Total %
Surgeons with experience in AM	12	7	65.5%
Surgeons with no experience in AM	7	3	34.5%

### Degree of usage of AM in Swedish hospitals

Of all the respondents, 20 surgeons were from the university hospitals, and 11 from non-university hospitals. Respondents from university hospitals cover all seven university hospitals in Sweden, labeled as UH1–7. All university hospitals in Sweden have adopted AM technology, and five of them have implemented AM technology in OMFS (Table 3). Further, UH1, 3, and 5 have more than 4 printers, whereas the remaining university hospitals have 1 to 3 printers each.

**Table 3:** Degree of AM usage in Swedish university hospitals

Hospital label	Adopted AM technology in the hospital	Adopted AM technology in OMFS	Number of printers owned
UH1	Yes	Yes	> 4
UH2	Yes	No	1–3
UH3	Yes	Yes	> 4
UH4	Yes	No	1–3
UH5	Yes	Yes	> 4
UH6	Yes	Yes	1–3
UH7	Yes	Yes	1–3

The survey covers surgeons from seven out of twenty Swedish non-university hospitals (including clinics), labeled here as NUH1–7. All seven hospitals have adopted AM technology, and six of them use AM technology in OMFS, as shown in Table 4. None of the non-university hospitals has more than 4 printers.

**Table 4:** Degree of AM usage in Swedish non-university hospital

Hospital label	Adopted AM technology in the hospital	Adopted AM technology in OMFS	Number of printers owned
NUH1	Yes	Yes	1–3
NUH2	Yes	No	1–3
NUH3	Yes	Yes	1–3
NUH4	Yes	Yes	1–3
NUH5	Yes	Yes	1–3
NUH6	Yes	Yes	1–3
NUH7	Yes	Yes	1–3

The result shows that out of the 14 investigated hospitals, all have implemented AM technology, and 11 out of 14 implemented AM technology in OMFS, making the adoption rate of AM in OMFS 78.57%. Further, the *p* value of the independent sample *T*-test is 0.23 which is higher than the significant level of 0.05 concluding that the university type does not have a significant effect on the adoption of AM in OMFS.

### AM solutions used

Descriptive and content analysis were used to answer the question of the AM solutions used in the hospital. Extracting answers from both multiple-choice questions (Q14–Q15) and open-ended question (Q16), three themes were identified: surgery types, type of AM used, and machine and software used. AM was mainly utilized in orthognathic and trauma surgeries. The most common AM technology in OMFS was material extrusion and vat polymerization. Only surgeons from the non-university hospitals mentioned specific machines and software used in AM production. The machines mentioned were small production capacity FDM and SLA machines. The software mentioned includes 3D slicer, Meshmixer, and GrabCAD. Interestingly, “self-developed software” was also mentioned by one respondent from a university hospital. Generally, university and non-university hospitals in Sweden use professional software and equipment, as indicated in Table 5.

**Table 5:** AM solutions utilized in OMFS in university and non-university hospitals

	AM solutions used in OMFS	Frequencies
Surgery types (*n* = 8)	Orthognathic	4
	Trauma	3
	Reconstruction	1
Type of AM used (*n* = 16)	Binder jetting	1
	Material extrusion	7
	Powder bed fusion	2
	Vat polymerization	6
Machine and software used (*n* = 7)	Professional equipment (i.e., printers)	3
	Professional software (e.g., 3D slicer, Meshmixer, GrabCAD)	3
	Self-developed software	1

Table 6 shows that surgeons in the university hospital used AM technology for all purposes, except biomanufacturing. None of the non-university hospital surgeons used AM technology for implants and tools, instruments, and parts for medical devices. Numerically, medical models and surgical guides and splints are the most selected purposes for AM in both university and non-university hospitals.

**Table 6:** Clinical areas of AM use in university hospitals and non-university hospitals (*n* = 19)

Clinical area	University hospitals (*n* = 12)	Non-university hospitals (*n* = 7)
Medical models	11	7
Implants	5	0
Tools, instruments, and parts for medical devices	1	0
Medical aids, supportive guide, and splints	9	6
Biomanufacturing^a^	0	1*

^a^No further information provided

### Accessing AM technology in hospitals

Altogether 13 surgeons answered the open-ended question (Q16) of how AM technology is accessed. Based on content analysis, it is visible that eight surgeons from university hospitals access AM solutions in-house and often have 3D printing engineers and technicians collaborating with the surgeons to plan, design, and print the product for the surgery. Only one university hospital surgeon responded to have access outside of the hospital. Non-university hospitals also have access to AM technology in-house. None of the non-university hospitals mentioned access outside of the hospital. In general, very few surgeons answered this question (Table 7).

**Table 7:** University hospital and non-university hospital access to AM technology

Access	University hospitals (*n* = 9)	Non-university hospitals (*n* = 3)
In-house	8	3
Outsource	1	0

## Discussion

AM is a promising technology with various applications in OMFS. Previous studies have demonstrated that OMFS and dentistry spearheaded AM adoption in clinical use. However, relatively few studies have explored the actual clinical adoption of AM in OMFS, and thus there is a weak evidence base concerning the adoption of AM in these areas. This study aims to shed light on this aspect by focusing on adoption in one innovative country, namely, Sweden. The study provides three important contributions relevant to both surgeons and hospital managers.

First, no difference between university hospitals and non-university hospitals was detected in terms of AM adoption. One would assume that non-university hospitals would be less innovative since they do not have tight bonds to a university and thus most likely invest less in R&D. In line with this, non-university hospitals focus more on providing routine care than on researching how to improve routine care. Taken together, it is less likely that non-university surgeons will come in contact with new technology such as AM. This assumption on a lower adoption rate of AM among non-university hospitals was validated in Pabst et al. [17] study on AM adoption in OMFS in Germany but not in the present study on adoption in Sweden. The present study covered all university hospitals but only a minority (7/20) of non-university hospitals. Thus, the surgeons that already work with AM might have been more prone to answer the survey leading to an overestimation of the adoption rate among non-university hospitals. However, based on our experience, some non-university hospitals commonly 3D print surgical guides for their routine orthognathic surgery and that type of surgery can be assumed to be well-represented in high numbers in these clinics. Also, the use of AM in clinical routine may be found in more advanced procedures handled mainly by specialists, which could be studied in future through a survey. Future research could also investigate further whether there are differences in the adoption of AM in OMFS between the university and non-university hospitals. This research should cover Sweden but also other countries to investigate if Sweden excels in adopting AM due to its innovative nature. Future studies could also cover the R&D budgets of the university and non-university hospitals and ties between surgeons and hospitals to further explore the possible high adoption rate of non-university hospitals.

Second, out of 29 surgeons who responded, 34.5% had never used AM technology before. This data is similar to German data, where 37.8% of surgeons had never used AM [17]. The Swedish AM agenda stated that the overall adoption rate of 3D printing technology in Sweden is lagging compared to other European countries, and the infrastructure used for research in Swedish universities is less advanced than in other parts of Europe [21]. This agenda concerned the adoption of AM in general. AM in OMFS exhibits a favorable exemption to this statement. The finding that more than 60% of OMF surgeons in Sweden and Germany investigated in the respective studies have adopted AM in OMFS enforces the assumptions of previous studies that AM in OMFS and dental surgery are leading the adoption of AM in clinical use [5]. When over 60% of users have adopted a certain technology, it can be perceived as a mature technology [22]. In future, it would be interesting to investigate the use of AM by OMF surgeons among private practices in Sweden. The scope of this activity is mostly centered around dental implantology, and one would expect extensive use of surgical guides. Whether AM adoption in OMFS shows a similar pattern in other OECD countries is still an empirical question for further research.

Third, despite the widely reported use of AM in OMFS in Sweden and the various clinical applications, it is still centered on less demanding applications such as medical models and surgical guides (which can be used for planning or training purposes). In contrast, more advanced medical device applications, such as instruments and parts, are still relatively rare. The fact that AM is not used in instruments and medical devices could depend on the complex regulatory climate in the EU, where new requirements are posed by both manufacturers and regulatory agencies [23]. For instance, private clinics and hospitals in French and Germany rely on professional external AM companies to help with production due to extensive costs and restrictions from relevant laws and regulations [17, 24]. Further, there are important differences between university and non-university hospitals regarding clinical applications. Five surgeons from the university hospital reported working with implants, compared to zero from non-university hospitals. This could indicate that university hospitals are still at the forefront of using new solutions in surgery, whereas non-university hospitals focus more on facilitating surgery planning through AM. However, one surgeon from a non-university hospital reported that they use AM in biomanufacturing compared to zero from university hospitals. This indicates that non-university hospitals could also be at the forefront of using AM, and thus more research is needed to investigate AM adoption in non-university hospitals.

Swedish university hospitals have in-house and outsourced solutions, where in-house is the primary production mode, and non-universities responded only have in-house solutions. Four university hospital respondents emphasized the importance of having skilled AM engineers, indicating that the use of AM technology in OMF is a complicated task requiring multiple professions’ cooperation. Although most hospitals indicate in-house access to AM, this finding should not be overly stressed since very few replied to this question. Given the regulatory hurdles and involvement of multiple professions in in-house manufacturing, one would assume that this is still relatively rare. Further studies, preferably qualitative interviews, are needed to explore how hospitals access AM and why.

## Conclusions

There is an indication that AM in OMFS is a mature technology. All the investigated hospitals have implemented AM technology, most of which also provide AM in OMFS. The most common use areas center on surgical guides and medical models. Orthognathic and trauma repair are the major surgeries that involve AM. The most utilized AM methods in OMFS are material extrusion and vat polymerization. Some limitations apply. First, the survey was voluntary, without mandatory answers to each item. This causes differences in data size in different items, especially the open-ended question. Complete data would be beneficial for a complete analysis, but this was a strategic risk since the population of surgeons is very busy and hard to reach. Second, it is more likely for surgeons who have strong feelings and opinions concerning AM to participate in this research. Conversely, surgeons who have never used or are unaware of AM will be less likely to respond. However, the data covered in this study also captured non-adopters and thus should be able to provide a comprehensive view of AM adoption in OMFS.

### Supplementary Information

Below is the link to the electronic supplementary material.

Supplementary file1 (DOCX 22 KB)

## Data Availability

The data supporting the findings of this study are available within the article and its supplementary material.
