# Supplementary material for: Adoption of additive manufacturing in oral and maxillofacial surgery among university and non-university hospitals in Sweden: findings from a nationwide survey
Source: Oral Maxillofac Surg. 2023 Mar 15;28(1):337–43. doi: 10.1007/s10006-023-01147-5 (PMC10914879; doi:10.1007/s10006-023-01147-5)
Supplement: Supplementary file 1 — Supplementary file1 (DOCX 22 KB) [file 10006_2023_1147_MOESM1_ESM.docx]

**Supplementary file 1.**

Adoption of additive manufacturing in oral and maxillofacial surgery among university and non-university hospitals in Sweden: findings from a nationwide survey

Oral and Maxillofacial Surgery

Xuewei Zheng (ORCID ID:)^1^, Ruilin Wang (ORCID ID:)^1^, Anders Brantnell (ORCID ID: 0000-0001-6841-7644)^1,2^* and Andreas Thor (ORCID ID: 0000-0001-9590-2039)^3^

^1^Department of Civil and Industrial Engineering, Industrial Engineering and Management, Uppsala University, Ångströmlaboratoriet, Lägerhyddsvägen 1, Uppsala, 752 37, Sweden.

^2^Department of Women’s and Children’s Health, Healthcare Sciences and e-Health, Uppsala University, MTC-huset, Dag Hammarskjölds väg 14B, 1 tr, Uppsala, 752 37, Sweden.

^3^Department of Surgical Sciences, Plastic & Oral and Maxillofacial Surgery, Uppsala University, Akademiska sjukhuset, ingång 79, SV, Uppsala, 751 85, Sweden.

*Corresponding author: Dr. Anders Brantnell

E-mail: [anders.brantnell@angstrom.uu.se](mailto:anders.brantnell@angstrom.uu.se)

| **Supplementary file 1. Questionnaire and response rate** | | | |
| --- | --- | --- | --- |
| Survey Question | | Response rate | |
|  |  | % | N |
| Q1 | What is your name? | 100% | 31 |
| Q2 | What is your age? | 100% | 31 |
| Q3 | What is your gender? | 100% | 31 |
|  | 1. Male 2. Female |  |  |
| Q4 | At which hospital do you work? (If retired, please specify) | 100% | 31 |
| Q5 | Is your hospital a university hospital? | 100% | 31 |
|  | 1. Yes 2. No |  |  |
| Q6 | What is your highest academic title? | 100% | 31 |
|  | 1. DDS 2. DDS, MD 3. PhD 4. Associate professor (Docent) 5. Professor 6. Other (Please Specify) |  |  |
| Q7 | Please select the following that best describes your current position  in the hospital. | 100% | 31 |
|  | 1. Specialist Oral and Maxillofacial surgery and DDS 2. Specialist Oral and Maxillofacial Surgery, DDS and MD 3. Resident (ST) Oral and Maxillofacial Surgery and DDS 4. Resident (ST) Oral and Maxillofacial Surgery, DDS and MD 5. Other |  |  |
| Q8 | Which kind of surgery do you do the most on a monthly basis? | 100% | 31 |
|  | 1. Dento-alveolar surgery 2. Dental implant surgery 3. Bone grafts and replacement 4. Orthognathic surgery 5. Maxillofacial trauma 6. TMJ surgery 7. Pathology and Reconstruction 8. Facial cosmetic surgery 9. Others |  |  |
| Q9 | Are you aware of additive manufacturing (AM) applications in oral and maxillofacial surgery? | 93.54% | 29 |
|  | 1. Yes 2. No |  |  |
| Q10 | Has your unit adopted AM technology in oral and maxillofacial surgery? | 93.54% | 29 |
|  | 1. Yes 2. No 3. Do not know |  |  |
| Q11 | Has your hospital adopted AM solutions in areas other than oral and maxillofacial surgery? | 93.54% | 29 |
|  | 1. Yes 2. No 3. Do not know |  |  |
| Q12 | How many 3D-printers does your hospital have? | 93.54% | 29 |
|  | 1-3  4-6  7 or more  4. Do not know |  |  |
| Q13 | Do you have any experience of using AM in your work? | 93.54% | 29 |
|  | 1. Yes 2. No |  |  |
| Q14 | What type of AM technology do you have experience of? | 58.06% | 18 |
|  | 1. Binder Jetting 2. Directed Energy Deposition 3. Material Extrusion 4. Powder Bed Fusion 5. Sheet Lamination 6. Vat Polymerization 7. Material Jetting 8. Do not know 9. Other (please specify) |  |  |
| Q15 | At which medical areas did you use AM technology for? | 58.06% | 18 |
|  | 1. Medical Models 2. Implants 3. Tools, instruments, and parts for medical devices 4. medical aids, supportive guide, splints and prostheses 5. Biomanufacturing 6. Do not know 7. Other (please specify) |  |  |
| Q16 | Please describe your experience of AM in your hospital | 41.93% | 13 |
